# Student researchers’ perceived prerequisites for voluntary research collaboration in the context of Flemish and Chinese universities

**DOI:** 10.1371/journal.pone.0197960

**Published:** 2018-05-24

**Authors:** Sisi Li, Chang Zhu, Shasha Li

**Affiliations:** 1 Department of Educational Sciences, Faculty of psychological and educational sciences, Vrije Universiteit Brussel, Brussels, Belgium; 2 Faculty of Law, Shanghai Maritime University, Pudong, Shanghai, China; Dalian University of Technology, CHINA

## Abstract

While numerous papers have illuminated the worthiness of research collaboration, relatively few have addressed its prerequisites. In our study, seven prerequisites for research collaboration were extracted from the existing literature, and 460 student researchers were surveyed for their perceptions of the prerequisites’ importance. Focusing on voluntary research collaborations rather than brokered ones, it was found that socially oriented prerequisites such as reciprocal interactions, accountability, trust, and equality are perceived of more importance than prerequisites of psychical proximity, networking channels, and funds and material supplies (substance- and entity-related prerequisites). With latent regression analyses, we also found that Chinese and older, more experienced researchers are inclined to stress the importance of equality. Researchers of different cohorts prioritise substance- and entity-related prerequisites disparately. Specifically, Chinese researchers emphasise the necessity of funds, while researchers from first-tier universities place more value on networking channels. Disciplinary differences for the prerequisite of proximity were also discovered. Based on these results, discussion and implications were referred. Further suggestions on research collaboration studies are rendered.

## Introduction

In recent decades, a great deal of literature has focused on scientific collaboration [1–4]. Generally speaking, most scientific collaborations focus on state-of-the-art technology and patents [5,6], which are usually in science, technology, engineering and mathematics (STEM) disciplines and are inter- or trans-disciplinary in nature. Though the merits of productive collaboration are broadly applied in various domains, such as the sciences, education (i.e. those who seek to improve the efficiency of middle schools), industry technologies and community service [7], universities serve as incubators for new knowledge and are the cornerstone of research collaboration [8].

In universities, research collaboration is believed to generate new ideas effectively by utilising the skills and knowledge of scholars from different disciplines [9,10], thereby conquering academic difficulties by converging breakthrough technologies and ideas [11] and improving research productivity [12,13]. Apart from that, improved self-esteem, interpersonal relationships and the spirit of teamwork are also rewarding outcomes that collaborative activities commonly produce [14,15].

Several opinions have been offered to clarify what research collaboration actually is. Katz and Martin [14], for example, tried to identify the social processes and boundaries of research collaboration, but they discovered that this concept has very ‘fuzzy and ill-defined borders’ (see page 8 in [14]). More recently, Leibowitz et al. [2] posited that collaborative research in universities can be conducted by both loosely working groups and tightly working ones, as long as they are beneficial to solving the problem [16]. Bozeman et al. [1] once defined collaboration as ‘a social process whereby human beings pool their human capital for the objective of producing knowledge’ (see page 3 in [1]). By this definition, the active participants involved are considered human capital, and their purpose is to produce knowledge, for which the whole process is socially related. This means the research collaboration is concerned with social relationships and social interaction. Thus, contrary to what is commonly believed, instead of being equal to the quantity of co-authorships in papers or patents, research collaboration does not necessarily take the form of co-authorship or even have any research output; sometimes, the ethical practices and power relations involved in group research may compromise the real contributions of such collaborations [17,18]. Therefore, realising that research collaboration is actually a social process achieved by people who are actively producing new knowledge, recent studies on research collaboration go beyond focusing on the extent to which the research collaboration can improve research output and productivity. Instead, recent studies concentrate on the power relations involved [5,19], explore the emerging social relations and examine the successful mechanisms of research collaboration [20,21].

There are three frontiers of social relations in research collaboration. First, based on the assumption that various researchers have different perceptions of research, Brew, Boud, Namgung, Lucas and Crawford [22] found that active researchers are more likely to engage in research team collaborations because they hold a trading view on research. By this, it means those researchers perceive the finished research output as the leverage in exchange for ‘money, prestige or recognition’, and the trading view is judged in terms of the ‘relationships with other people’ through the research collaboration process (see page 281 in [23]). Second, in contrast to the commonly held view that bureaucracy impedes research in universities [24], Shrum, Genuth and Chompalov [25] argued that bureaucracy is actually an institutional assurance for larger and longer-trusted research collaborations. Because even though the sense of trust in research collaboration is inversely related to conflicts, the presence of bureaucracy in an organisation can minimise the negative consequences of conflicts and improve the stability of relationships among research collaborators. In this sense, the conflicts in brokered research collaboration do not necessarily obstruct research collaborations. Third, the conflicts and competition that occur in the process of research collaboration, as well as the social roles played by the collaborators, have been a long-debated topic among researchers. For example, Saari [4] elaborated on the communication process between Finnish and American research groups and detailed the group dynamics, showing that relationships among research collaborators could be particularly vulnerable when the group members were free of bureaucratic restrictions. Kezar [21] thought that an institution could promote research collaboration through three developmental stages: commitment building, commitment fulfilment and commitment sustainment. Both Saari [4] and Kezar [21] explained the prerequisites for ensuring a smooth research collaboration process, such as understanding how to balance the relationships among the collaborative parties and how to expand and sustain collaborative social roles and relations.

Although many insightful views have been presented on the social relations of research collaboration, most prior research has concentrated on brokered research collaboration in inter-organisational scenarios [5, 6, 9, 12]. Therefore, research collaborations conducted among individual researchers on a volunteer basis still require exploration. Building on the existing literature, our study explores how researchers generally perceive the preconditions of research collaboration rather than centring on the collaboration itself. In this way, it fills the gap in scholarship by discovering how researchers prioritise research collaboration requirements and how social relations are involved in that process.

## Research collaboration prerequisites: An analytical framework

In line with the three frontiers of social relations in research collaboration that were defined by prior studies, the prerequisites highlighted in our study also examine the social relations throughout the research collaboration process. To be specific, our study investigates the prerequisites of research collaborators in terms of ‘relationships with other people’ (see page 281 in [23]). Jeong, Choi and Kim [26] investigated the determinants for different research collaboration modes and found that informal communication, cultural proximity, academic excellence, external fund inspiration and technology developmental levels are all crucial factors. Our study, however, excludes determinants that differ from research collaboration modes, including academic excellence and cultural proximity. Because the requirement of academic excellence or academic competence is implied when planning a research collaboration, so these determinants are not considered in our study. Similarly, cultural proximity is not considered because our research does not specifically examine international research collaboration.

### Substance- and entity-related prerequisites

Careful inspection of the five determinants of the research collaboration modes identified by Jeong et al. [26] provides insight into their prerequisites. As most research collaborations are initiated among known contacts, informal communication is a key starting point to recruit potential collaborators [14]. However, physical proximity is a more suitable prerequisite for research collaboration than informal communication, since informal communication could perceptually mean research collaboration. Furthermore, by extending the concept of informal communication in correspondence to ‘physical proximity’, the prerequisite of ‘networking channels’ (which provide potential researchers with a basis for preliminary and subsequent contact), is also of vital importance [27,28]. Additionally, instead of interpreting funds and supplies as purposes for research collaboration, these are more appropriately interpreted as another prerequisite for research collaborators—since those researchers are more inclined to be productive if resources such as funds are provided from the start [29].

#### Physical proximity

Physical proximity is defined as geographical closeness or being ‘on the scene’, which yields ‘a high level of casual, serendipitous, spontaneous, nonintrusive communications’ (see page 14 in [30]). Kraut, Fussell, Brennan and Siegel [31] also found that physical proximity has a positive effect on research collaboration: by encountering each other more often, researchers are more inclined to share psychological intimacy, emotional attachment and common research interests. As such, those who are physically close to each other are more likely to have high quality research outputs than those who collaborate from a distance, even though distant communication measures are abundant.

#### Networking channels

Networking channels, which bear many similarities to telecommunication systems, serve as a transmission medium for communication, because they can provide the occasions, measures and techniques needed for potential collaborators. It is argued, by Goel & Grimpe [32], that networking channels can be differentiated into active ones and passive ones, the former refers to conference attendance, the latter is mainly brought by the existing contacts or contacts induced by taking structured degree courses. Compared with small universities, larger ones can provide more choices of potential collaborators when transmitting knowledge [33]. This view was also reinforced by Duysburgh et al. [12]. They indicated that separating the attachments between junior workers and technical groups would result in a lack of networking channels, which would be detrimental to future collaboration. Nevertheless, various measures and techniques can be applied to networking channels. For instance, Internet-based networks, which can serve as a supplement for seminars, workshops and conferences, are believed to be the most extensively applied method of amplifying an existing network of contacts, leading to more chances for research collaboration [34]. In addition, Kyvik & Reymert [35] contended that there are disciplinary differences in network channels, for which the domain of medicine, health, and natural sciences, according to them, could be more likely to rely on it.

#### Supplies and funds

In recent years, research funding has played a fundamental role in improving the research production of academics, especially for those in technical and natural sciences, because expensive equipment is needed to conduct daily experiments [36]. Given the importance of supplies and funds, some suggest that funding is the primary motive for collaboration when looking for a potential collaborator [12]. Goel & Grimpe [32] found that funding tends to be a determinant for collaboration, in the sense that active networking like conference attendance need funds to support. Related to the prerequisite of networking channels, funding provides researchers with greater research mobility and more opportunities to meet new contacts, which are essential for planning further research collaborations [37].

### Socially oriented prerequisites

Though substance- and entity-related prerequisites such as physical proximity, networking channels, and supplies and funds are indispensable for research collaboration, successful collaboration requires more than these. The patchwork quilt metaphor proposed by van Swet, Armstrong and Lloyd [38] delineates the research collaboration process. The starting point of this process is an ‘agreement’; the collaborators then ‘negotiate’, ‘shar[e] ideas and progress’, ‘ask for’ advice and ‘make decisions and adjustments’ (see page 649 in [28]). Similar to the prerequisites for the patchwork quilting process, the characteristics of being reciprocal, or having a ‘fair trade’, and being accountable (equivalent to shared privilege and empowerment, respectively), as well as the requirements of trust and equality, are fundamental to the research collaboration process [39]. The same observations are found in research collaborations across borders [9] and across sectors [7,40]. As most of these prerequisites are concerned with people and their relationships, they are referred to as socially oriented prerequisites.

#### Reciprocal interactions

Reciprocal interactions are two-way, mutual and ability-based. In the scenario of university-community research collaborations, mutually rewarding and valued relationships are central to the collaboration partnership [7]. As is evident from interdisciplinary collaborations and technique exchanges stemming from cross-institute collaboration occasions, collaborators only find value in (and feel motivated to engage in) collaboration if it is reciprocal [7,14]. Molm, Melamed and Whitham [41] also showed that reciprocal exchanges improve the bonds of attachment, the relational climate of trust and solidarity among researchers.

#### Trust

In the context of research collaboration, trust is defined as believing in the reliability, competence, openness and honesty of the researching partners [39]. Based on that trust, knowledge can be developed, shared or applied through the collaboration process [42]. Moreover, on the path from knowledge development to knowledge sharing and application, being trustworthy is fundamental and fosters a sense of safety. Being trustworthy can also allow one to foster academic achievements and maintain long-term collaborative relationships with one’s collaborative partners.

### Equality

Equality is defined as ‘individuals or groups of individuals being treated fairly and equally and no less favourably’ [43]. People from different cultures perceive the importance of equality differently based on their perceptions of how power should be distributed [44]. In fact, collaborators are not necessarily equal in their titles and notoriety throughout the brokered research collaborations, namely the collaboration that are mediated by a third party. For example, Lin & Hsu [45] claimed that there is no equality in scientific and engineering research collaboration because experienced researchers’ opinions are considered more important than those of less experienced researchers. However, if the research collaboration is not conducted within a restrictive bureaucratic framework—for instance, if it is conducted on a voluntary basis—inequality could then cause problems. In this vein, equality is an essential prerequisite for potential collaboration [2].

### Accountability

Pluralism, professionalism and consumerism, which respectively referred as external accountability, internal accountability, and client accountability, were considered as the origins of accountability of the large voluntary organizations[46]. In research collaboration, accountability is the state of being responsible for the work. Since ‘to talk of accountability is … to talk of control’ (see page 146 in [47]). Our study holds the view that collaboration partners should take responsibility for their own knowledge authenticity and objectivity, thus being responsible for the production of new knowledge [48].

Even though seven prerequisites—physical proximity, networking channels, supplies and funds, reciprocal interactions, trust, equality, and accountability can be extracted from the existing literature, people perceive the prerequisites for research collaboration differently because of their individual cultural and academic backgrounds [49]. For example, research networks supporting shared language and vision are assumed to be the prerequisites for educators’ research practices [50], and Bozeman and Corley [51] found that access to larger grants is the first prerequisite for scientists’ research collaborations. In this sense, examining how individual researchers identify the prerequisites for potential collaborations could be very illuminating, at both theoretical and practical levels.

## Research assumptions and questions

Altogether, limited by fragmented research samples and varying assumptions, researchers have settled on different conclusions for the prerequisites of research collaboration in the existing literature. Lee, McCauley and Draguns [52] found that, broadly speaking, researchers’ characteristics vary based on their nationality, and differences in researchers’ demographical and research backgrounds could explain their divergent perceptions of research collaboration. Uhly, Visser and Zippel [53], for example, indicated that a ‘glass ceiling’ exists for female academics because they are impeded by family arrangements. It means that female academics are less likely to engage in international research collaborations. However, empirical studies found that female scientists are more inclined than male scientists to engage in interdisciplinary collaboration [54] as well as general research collaboration [51]. In addition, Van Rijnsoever and Hessels [54] thought that interdisciplinary collaboration occurs more often in strategic rather than basic disciplines. The former refers to the disciplines that have wide application in the real life, such as medicine and human geography; while the latter are concerned with the disciplines that act as the theoretical basis for common knowledge, for example, mathematics. In contrast, Fox et al. [55] unearthed that female researchers would be less likely to engage in international collaboration. Furthermore, researchers in the domain of engineering, medicine and biology rely more frequently on funds than those in other disciplines [14]. Existing scholarship also explains why researchers in well-developed educational systems collaborate more often than those in developing ones and why researchers in English-speaking regions collaborate more often than researchers in non-English-speaking regions [10]. Additionally, individuals with more work experience are more inclined to collaborate across disciplines [54]. So, would such differences in the inclination towards research collaboration cause discrepancies in the way researchers judge its prerequisites? Also, since university rankings are based on research output, and research collaboration is found to promote research output [1], would students affiliated with better-ranking universities have different perceptions of the prerequisites for research collaboration compared with those of lower-ranking universities?

**Fig 1:**
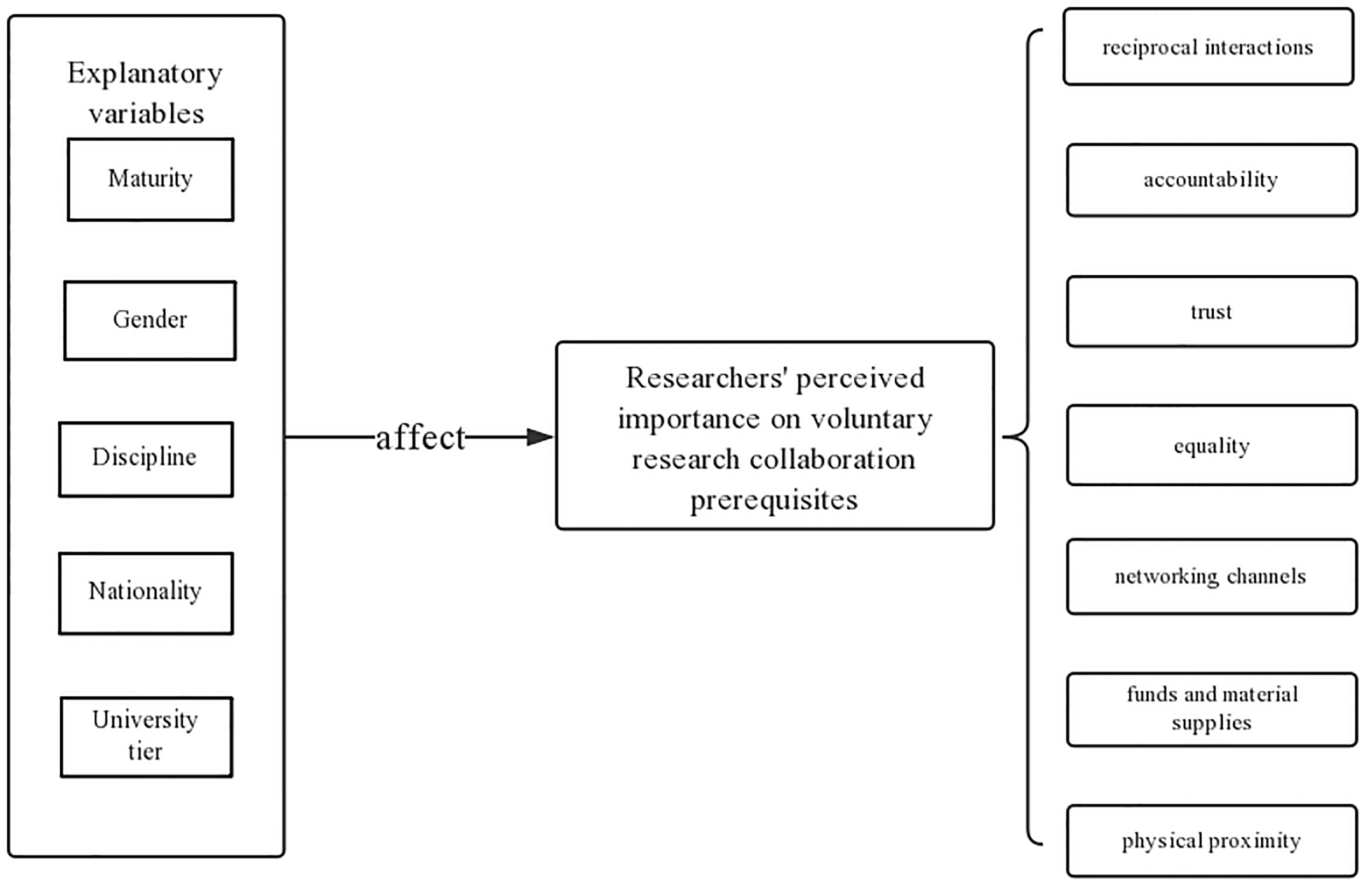
Assumption that different nationalities, disciplinary domains, maturity levels and university rankings could affect researchers’ perceptions of the prerequisites for research collaborations.

Thus, as is shown in Fig 1, our study explores how student researchers perceive the seven prerequisites of research collaboration (Research question one, hereinafter noted as RQ1). Also, we want to examine whether nationality, disciplinary domain, maturity level and affiliated university ranking affect the researcher’s perceptions of the prerequisites for choosing a research collaborator (RQ2). Apart from that, we also intend to discover how the aforementioned factors could influence the way researchers perceive the prerequisites for collaboration (RQ3).

## Design and methodology

Our study has three areas of focus. First, we think voluntary collaboration is different from the brokered one. Chompalov et al. [56] examined 53 multi-institutional collaborations in physics and allied sciences. According to the degree of bureaucratization, formalization, hierarchy, scientific leadership, and division of labour, four collaboration types are emphasized. Those collaboration types are respectively bureaucratic, leaderless, non-specialized, and participatory collaboration. What described as participatory collaboration is actually the collaboration that is with low bureaucracy, no strict formalization, and no obvious leadership or hierarchy, but is initiated and processed by freewill (voluntary research collaboration). Since collaboration organizing style differences practiced by different researchers (tenured or untenured) could entail some underlying motivational, behaviour, and perception variations [22,25,57,58], to highlight this point, we research on how researchers perceive voluntary research collaboration prerequisites.

In line with what above- mentioned, because tenured researchers might not consider collaboration prerequisites the same way as student researchers, we specify our concentration on student researchers. Benchmarked on the definition of ‘research career structure’ identified by the European Commission [59], we centre on researchers of early career profile, who are essentially characterized by the career stage up until their PhD. Because on the one hand, even though those early career researchers are less experienced as researchers, their contributions to academic outputs are quite optimistic [60]. According to Larivière [61], PhD students have contributed one third of the academic publications in Quebec, Canada. One the other hand, retaining and caring for the researchers that are in the ‘apprentice’ stage in the academia has always been the central topic in higher education, which makes our research meaningful [62,63].

Third, the study sample included early-career researchers of Flemish (Belgium) and Chinese because we wanted to examine whether different cultural origins affected the emphasis researchers put on research collaboration prerequisites. According to Earley [64], this two regions may in representation of two typical different cultures—individualism of the West versus collectivism of the East. Furthermore, researchers’ culture origin might influence their underlying perceptions. For example, Korean researchers, in whatever disciplines, are found to collaborate in a cohesive network [52], however, such effect is only found positive among westerners [35].

Based on the assumption that researchers’ perceptions are varied from different nationalities, disciplinary domains, maturity levels and university rankings, we applied a latent class analysis (LCA) to examine which of those factors was the most decisive. In this way, we test whether there were differences in the degree to which those factors affected the researchers’ perceptions of research collaboration prerequisites. Latent class regression is appropriate when the variables are multivariate and categorical in nature. What’s more, the latent ‘class’ is featured when a pattern of conditional probabilities could be indicated for the variables to take on certain values [65].

### Participants and data collection

To explore our research questions empirically and to comply with the research design, student researchers holding degrees at post-bachelor levels were surveyed. Specifically, the participants are from four research-intensive universities in Flanders (Belgium), and Chinese student researchers from ten research-intensive universities in north-western, southern and eastern China were selected for the sample. Among the ten Chinese research-intensive universities, six are ‘985 institutions’ (i.e. first-tier universities in China), while the other four are ‘211 institutions’ (i.e. second-tier universities in China). Of the four Belgian universities, two are at the comparable ranking level as ‘985 institutions’, hence is regarded as first-tier university in our study, the same way, the other two are classified as second-tier. Thus, the sampling not only met the variability and representation requirements, but it also maintained a good balance for the sample size of each subgroup [66].

In addition, the explanation and example for each prerequisite are exemplified in the survey paper, thus to assure the participants understand them accurately. Before the survey was initiated, the potential participants were informed of the purposes and objectives of the research. They were told that they could refuse to be surveyed if they thought the survey items did not correspond with their research experience or if they were uncomfortable with it for any reason. In the surveying process, student researchers indicated that they ever have had any research collaboration on a voluntary basis were invited to rank each of the seven prerequisites on a scale from most important (7) to least important (1). Furthermore, to facilitate the survey responses, we wrote in our survey that we will not disclose the data to any third parties, and the participants can trust us for the data confidentiality. Thus, demographic and research background data of participants were also collected, and the identity of the participants remained anonymous. Altogether, 476 survey responses were collected through the data collection process. After the uncompleted surveys were eliminated, 460 were identified as valid. The demographic profiles of the research participants are displayed in Table 1.

**Table 1:** Demographical profile of research participants.

Variables	Categories	Frequencies	%
Sex	Male	226	49.1
	Female	234	50.8
Nationality	Chinese	248	53.9
	Belgian Flemish	154	33.4
	others	58	12.6
Working year	“year< = 2”	211	45.8
	“3<year<5”	129	28
	“year> = 5”	120	26
Education level	Master researchers	161	35
	PhD researchers	299	65
Disciplines	Social sciences humanities	226	49.1
	STEM. & Bio and Medicine	200	43.4
	Others	34	7.3
Research output	“published”	221	48
	“have not published”	239	51.9
Age	“up to 30”	312	67.8
	“above 30”	148	32.1
University tiers	1st tier	135	29
	2nd tier	248	53.3
	Others	82	17.6

### The perceived importance on seven collaboration prerequisites (Dependent variables)

As our study sought to measure the perceived importance of seven research collaboration prerequisites, participating researchers were asked to indicate how important they considered each of the seven prerequisites for research collaboration. For each of the prerequisite, ranking it as ‘1’is referred to the least important and ‘7’ the most important prerequisite. The participants were told to rank each prerequisite (altogether 7 prerequisites) as exclusive as possible, tailoring to the ranking scale (1–7), because variability was required to detect their perceived preferences.

### Explanatory variables

According to the research assumptions, the researchers’ maturity level, gender, disciplinary background, university tier and nationality were collected as explanatory variables for differences in their perceptions of the prerequisites for research collaboration (as is shown in Fig 1). Because the variable of work experience is related to social experience, it is labelled ‘social experience’ herein [67]. Moreover, ‘whether or not the researcher has published a paper/papers’ is an indicator of the researcher’s academic maturity level. According to Bozeman and Slade [1], a high maturity level enables the pooling of human capital and fosters the development of more network ties. Therefore, ‘working experience’, ‘whether or not the researcher has published a paper/papers’, ‘educational level’, and ‘biological age’ are all collected as participants’ background information. Those information is supposed to serve the predictor of maturity, and they may generally covariates for each other; as researchers are inclined to have more social experience and become more academically mature as they spend more time on academic activities.

### Data analysis

In this study, latent class analysis (LCA) and latent regression analysis were applied to answer the research questions. Because LCA is based on cross-tabulated data, in which dichotomous or categorical variables with no more than four categories are the most manageable for inputting data cells [68], our input data had to be adapted in this manner. One advantage of LCA is that it does not hold many restrictive assumptions about model building [69]. Furthermore, it can ‘aggregate the responses and decompose the tabular frequencies into a set of classes or segments that [display] certain characteristics’ (see page 170 in [70]), which is fitting for both our research questions and our study design. In LC modelling, logical reasoning can occur based on the categorical data, with the assumption that an empirical relationship between an explanatory variable and a dependent variable can be classified (which can generate local independence). Moreover, LCA can also identify mutually exclusive LCs based on cross-tabulated data [71]. Based on the logic that explanatory categorical variables are conditionally dependent, interrelated and able to predict hidden classes in the general population, cohorts in the sample were divided into segments. In LCA, values from model fit indices, such as the Bayesian information criterion (BIC) and the Akaike information criterion (AIC), are more important indicators than p-values when choosing the most fit model [69]. In a latent regression model, within-class errors and R-squared values show how well the model can predict the accuracy of the classification and to what extent the latent regression model can explain the classes successfully, respectively [72].

## Results

### Socially oriented prerequisites are of more importance than most researchers think (RQ1)

Table 2 illustrates the participants’ perceived importance on research collaboration prerequisites. The results show that all the socially-concerned prerequisites were considered important when viewed as a whole. Specifically speaking, ‘reciprocity’ was perceived as the most important prerequisite (mean = 5.611, mode = 7.0), followed by ‘trust’ (mean = 5.442, mode = 6.0), ‘accountability’ (mean = 5.398, mode = 6.0), ‘equality’ (mean = 4.991, mode = 6.0), ‘networking channels’ (mean = 4.600, mode = 5.0) and ‘supplies and funds’ (mean = 4.574, mode = 6.0), while ‘physical proximity’ (mean = 3.377, mode = 1.0) was perceived as unimportant.

**Table 2:** The perceived importance on each prerequisite.

Prerequisites on collaboration	Observation	Mode	Median	Mean	SD
Trust	462	6	6	5.442	1.33
Equality	465	6	5	4.991	1.449
Reciprocal interactions	460	7	6	5.611	1.414
Accountability	460	6	6	5.398	1.482
Supplies and Funds	460	6	5	4.574	1.742
Networking Channels	443	5	5	4.6	1.774
Physical Proximity	443	1	3	3.377	1.95

### Researchers of different cohorts prioritise substance- and entity-related prerequisites differently (RQ2 and RQ3)

As the model summary of the LCA shown in Table 3 demonstrates, ‘reciprocity’ (R^2 = 0.042), and ‘accountability’ (R^2 = 0.062) do not hold clusters that are appropriate for representing disparate cohort perceptions, meaning that no LC is embedded in these prerequisites. However, ‘equality’ (BIC = 1839.262, R^2 = 0.697), ‘funds’ (BIC = 1851.717, R^2 = 0.692), ‘channels’ (BIC = 1695.478, R^2 = 0.620) and ‘physical proximity’ (BIC = 1707.356, R^2 = 0.611) hold more fitting BIC and R-squared explanatory values compared with their non-clustered counterparts. Hence, there are LCs implicit in those prerequisites. As such, the prerequisites of equality, supplies and funds, networking channels and physical proximity were further processed to detect how many LCs each prerequisite has. As seen in Table 3, equality, funds, networking channels and physical proximity can all be classified latently. Nevertheless, even though the prerequisite of ‘trust’ can be statistically classified into two LCs, the R-squared value (13.3%) indicates that the explanation ability of ‘LC1’ is extremely low. Additionally, the key predictor of the AIC and the BIC is a better fit when this prerequisite is not classified as two LCs (BIC, AIC (one latent class) < BIC, AIC (two latent classes)). Therefore, we have exempted the prerequisite of ‘trust’ from being classified into more than two LCs.

**Table 3:** Latent class analysis model summary on seven collaboration prerequisites.

Dependent variables	Number of Clusters	LL	BIC(LL)	AIC(LL)	AIC3(LL)	L2	DF	p-value	Class Error	R^2
Trust	2	-680.075	1573.896	1430.150	1465.150	510.845	414	0.001	0.120	0.442
Reciprocal interactions	1	-696.920	1497.622	1427.840	1444.840	461.297	431	0.151	0.000	0.042
Equality	3	-729.295	1839.262	1582.589	1644.589	1042.239	402	< 0.0001	0.187	0.697
Accountability	1	-727.949	1547.503	1485.898	1500.898	415.561	434	0.732	0.000	0.062
Supplies and Funds	3	-773.183	1851.717	1646.366	1696.366	542.685	399	< 0.0001	0.165	0.692
Networking Channels	2	-711.995	1695.478	1513.990	1558.990	1094.445	372	< 0.0001	0.059	0.620
Physical Proximity	2	-717.934	1707.356	1525.868	1570.868	1107.992	372	< 0.0001	0.070	0.611

In consistent to the latent class analysis, we acknowledged that only the collaboration prerequisites on equality, networking channels, funds and material supplies, and physical proximity could be further processed for latent regression analysis. As the latent regression analysis summarized in Table 4, the three class proportions for equality are 0.229, 0.953 and 0.967. The class proportions for funds are 0.260, 0.718 and 0.906; the class proportions for networking channels are 0.368 and 0.850. Finally, the class proportions for physical proximity are 0.270 and 0.713. The overall goodness-of-fit, which is above 60% for every prerequisite, is statistically and realistically acceptable, indicating that the modelling solution is stable. It should be noted, however, it is the segment that accounts for the largest proportion that primarily decides the characteristics of the model [70,73]. According to those ideas, ‘usually, large latent classes tend to share even larger proportions after the assignment, and small latent classes share even smaller proportions, possibly resulting in such contradictory outcomes as no single respondent being assigned to the small latent classes despite their presence’ (see page 1704 in [73]). Since the aim of our study is to investigate in which way predictors such as age and education level influence early-career researchers’ perceived importance on the prerequisites for research collaboration, the beta values that are congruent among the largest proportions of segments are highlighted in the Table 4.

**Table 4:** Determinants on collaboration prerequisites (Latent class regression results summary).

	Equality (overall R^2^ = .697)			Funds and supplies (overall R^2^ = .692)			Networking channels (overall R^2^ = .620)		Physical proximity (overall R^2^ = .611)	
	Class 1	Class2	Class 3	Class 1	Class2	Class 3	Class 1	Class2	Class 1	Class2
Class size(R^2^)	0.229	0.953	0.967	0.260	0.718	0.906	0.368	0.85	0.27	0.713
Gender								-.983(1); .983(2)		
Nationality		5.072(1)	8.113(1)		2.345(1); -.893(2)	4.070(1); -2.482(2)				
Working experience		-4.531(2)	-1.364(2)							
Age		-9.233(1); 9.233(2)	-0.426(1); 0.426(2)							
Education level		-3.427(2); 2.787(1)	1.989(1); -2.768(2)		-2.003(1)	-2.109(1)				
University tier					-.413(1); .135(2)	-3.686(1); 2.093(2)		1.603(1); -.113 (2)		
Discipline		-1.534(1)	-3.115(1)		-1.282(1);	-1.723(1)				.531(1); -1.017(2)
Research paper										-.579(1);.579(2)

Notes of coding: Gender (1): male, (2): female; Nationality (1): Chinese, (2): Belgian Flemish; Working experience (1): less than 2 years, (2): more than 2; Age (1): less than 30, (2): more than 30; Education level (1), master level, (2): PhD level; University tier (1): first tier, (2): second tier; Discipline (1): social sciences and humanities, (2): STEM and Bio, medicine; Research output (1): have published output, (2): do not have.

The beta parameter for each segment, which measures the ratio likelihood of the segment on the corresponding predictor category, is illustrated for every prerequisite. For example, as shown in Table 4 for the effects on perceiving ‘equality’, the beta effect suggests that ‘segment 2’ is influenced in a negative way by researchers with a PhD-level education (beta = -3.427) and in a positive way by researchers with a masters-level education (beta = 2.782). This means that PhD student researchers are not likely to prioritise equality as a prerequisite for their collaborators to the same extent as masters-level researchers. Likewise, segment 3 of ‘equality’ converges with segment 2 regarding the influence of education level. Additionally, the prerequisite of equality is positively influenced by the characteristic of age above 30 (beta (segment. 2) = 9.233; beta (segment 3) = 0.426) and is negatively influenced by age “up to 30” (beta (segment 2) = -9.233; beta (segment 3) = -0.426). What’s more, Chinese nationality induced a positive effect perceiving equality for collaboration (beta (segment 2) = 5.072; beta (segment 3) = 8.113). Apart from that, the effect of working experience, discipline, and age also inflict an influence for student researchers on the proclivity of equality for collaboration.

The same way, Table 4 shows the LC for supplies and funds. The estimated beta effect, which is shown in the column labelled ‘segment 1’, suggests that this segment may not strongly influenced by any factor. However, in the segments 2 and 3, which hold the largest R-squared values for that prerequisite, the prerequisite for funds and material supplies is positively influenced by Chinese nationality (beta (segment 2) = 2.354; beta(segment 3) = 4.070), negatively influenced by Flemish Belgian identity (beta(segment 2) = -0.893; beta(segment 3) = -2.482). Also, the tier genre of university also has an influence: on the one hand, second-tier universities are more likely to attach importance to funds and supplies (beta(segment 2) = 0.135; beta(segment 3) = 2.093); on the other hand, the first-tier university are less likely than their counterpart in the first-tier(beta(segment 2) = -0.413; beta(segment 3) = -3.686).

As for the perceived differences of networking channels as a prerequisite for research collaboration, which has two class segments, segment 2 presents a relatively strong positive influence on first-tier universities (beta = 1.603). Also, gender seems to affect researchers to perceive the networking channels, in particular, male researchers are more likely to devalue it as a prerequisite for collaboration(beta = -.983). The other factors did not affect the classification differences, either because the beta value was not significant or because the factor was not significant with a p-value larger than 0.05. As to the perceived importance of physical proximity, no specific factor influenced segment 1, but in segment 2, it was noted that disciplinary factors within STEM disciplines had a beta value equal to -1.017. Also, those who has published research would not think highly on physical proximity(beta = -.579).

## Discussion and implications

### Voluntary research collaboration requires different prerequisites in comparison with brokered one

The recently published work by Tsikerdekis & Yu [74] considered the physical proximity and funds as important environmental factors influencing researchers for intra-university research collaboration. However, without specifying it was brokered or voluntary collaboration, this study has very likely produced a ‘significant’ result that reflects the collaboration practice of the mixed composition of sample, since almost 70% of 352 researchers they investigated are tenured ones. Our results considered the collaboration organizing style and composition of sample, and specifically investigated voluntary research collaboration. We found that reciprocity, trust and accountability are normally considered the most essential prerequisites for research collaboration. There are several explanations for this. First, these prerequisites are highly concerned with the perceived social relations of a collaborator, and only after those requirements are provided can potential collaborators truly engage in collaborative activities. Second, if a researcher already has the conditions of physical proximity, funds and networking channels, the possibility of finding potential collaborators who are reciprocal, trustworthy and accountable, as well as showing equality, is relatively more important than if the researcher does not have these favourable conditions. This finding supports the view that research collaboration is a social phenomenon and reveals that there are power relations among collaborators [5,22].

Of all the prerequisites, reciprocity has the highest mean value, which is consistent with Emerson’s [75] claim that social relations commonly entail ties of mutual dependence. Furthermore, according to Kabanoff [76], the sense of equality is closely related to reciprocity. Thus, ‘equality’ and ‘reciprocity’ are two central prerequisites for research collaborators, and only after these two prerequisites are satisfied can trust and accountability be built. This detection reminds us the difference between voluntary research collaboration and the brokered one. In circumstances of voluntary research collaboration, successful research collaboration requires a stronger sense of ‘equality’, ‘reciprocity’, ‘trust’ and ‘accountability’ among the collaborators. However, brokered research collaboration may not require such strong personal relationships among collaborative parties because of the function of bureaucracy [25]. Based on this finding, it is possible that researchers who only perform voluntary collaboration will follow a different daily agenda, from those who practice mainly brokered collaboration. For example, voluntary collaboration behaviours may embed in many day-to-day interactions with the collaborate partners, while the brokered collaboration behaviours may include a lot of daily routine, hence is more stabilized. Also, it might be possible that voluntary collaboration may serve the pre-stage for brokered collaboration. For instance, researcher mobility experience induced by participating in brokered collaboration, may derive new opportunity for voluntary collaboration. Future research might consider to differentiate the two collaboration genres, thus identifying the practice patterns of them. Moreover, how the lived experience of voluntary collaboration behaviour helps to engender brokered collaboration is also of interest for future research.

### Perceived voluntary collaboration prerequisites are wield by strong cultural and educational contextual factors

Culture origin is a reason for researchers to perceive the collaboration prerequisites differently. For example, Chinese researchers stressed the requirement of ‘equality’ for choosing a research collaborator, while the Flemish cohorts are less so. This contradicts Hofstede et al. [44], for which, they thought that Asian people do not place as much value on equality. Confusing as it may seem, this phenomenon is somewhat consistent with a local study in China, in which Wang [77] found that Chinese researchers differ from other professional Chinese cohorts because ‘academic research groups’ become more sensitive to equality as their educational levels increase. Also, cultural differences coupling with other factors, might induce researchers to value differently on collaboration prerequisites. As the famous paper written by Kabanoff [76] indicated that ‘as the power difference in an organizational relationship increases, the psychological orientations of both parties tend to reflect a greater, though not necessarily equal, acceptance of equity as the distributive rule’ (see page 426 in [76]). It might be very appealing to investigate how culture origin differences affect the preference of equity or equality.

In the domain of education, biological feature like age is at odds with the educational related factors in determining people’s perception. On the one hand, age is positively related to the extent to which equality is required in social interactions [78]. On the other hand, PhD students do not consider the prerequisite of ‘equality’ to be as important. One explanation for this could be that PhD students are required to conduct as much research as possible before graduating [79], so it is possible that the other prerequisites take priority. Moreover, academic maturity and social maturity seem to align with each other in indicating researchers’ perceptions on equality. To be sure, more social experience would render people to perceive research in a ‘trading view’[22, 23], rather than to perceive ‘equality’ as a priority. But would researchers change their perceived importance on equality when they involve more intensively in brokered collaboration? This is an interesting point for further study to explore—would hierarchical brokered research collaboration change the perceived value on equality among researchers?

Also, educational contextual factors are hinted for researchers’ perception differences. Compared to Flemish (Belgian), Chinese researchers put more emphasis on the prerequisites of ‘funds’ and ‘material supplies’. There are two possible reasons for this. First, Chinese researchers are generally underpaid compared with other vocations in China. According to a local Chinese source, academic researchers in China cannot afford permanent accommodations in a major city based on their salary [80]. Second, the Chinese government does not invest much in the education system [81]. The same way, ‘not-sufficiently-invested’ second tier university may also make the researcher value more importantly on funds and supplies, in comparison to the first-tier. However, it might be tempting to think, that the perceived importance on prerequisites may not follow a ‘more insufficient, more important’ rule, but follow a need-basis. This may also be the reason that master student researchers and researchers in humanities and social sciences devalue the importance on supplies and funds when compare with STEM-disciplined researchers.

Regarding networking channels, researchers at first-tier universities are more likely to value them. This is somewhat consistent with the viewpoint of Chirikov [82], who argued that large, top-tier universities hold more networking channels, which makes the researchers more inclined to seek opportunities from those channels. On the prerequisite of ‘physical proximity’, however, researchers in STEM disciplines, biology and medicine do not emphasise the importance of physical proximity as much as researchers from other disciplines. This finding complements previous conclusions that researchers in STEM, biology and medicine are more likely to collaborate as a team, so researchers of those disciplines are less likely to let a lack of proximity impede their choice of a potential research collaborator [25, 51].

The research also leaves a few findings that cannot be directly explained by current literature. For example, why would researchers in social sciences and humanities value less importantly on equality? Why would researcher that had published output value less importantly on physical proximity, while those who had not publication value more importantly on it? We surmise disciplinary culture and the publishing experience may provoke some perception differences on how researchers perceive the general research collaboration process. Nevertheless, due to the thin literature on our topic, further study on such research topic might bring more light on such phenomena.

### Policy implications

Based on our findings, the difference between voluntary research collaboration and brokered one renders important policy implication. In a brokered research collaboration, the final goal is to serve the organisation, but when research collaboration is purely voluntary, the social relations are free from the ‘necessary evil of bureaucracy’ (see 117 in [25]), thus assigning importance to ‘reciprocity’ and all other socially oriented prerequisites before research collaboration is initiated [25]. In this sense, different from the brokered research collaboration that occurs in large organisations, research collaboration on a voluntary basis is hard to sustain and fulfil. Generally speaking, in a scientific context, bureaucracy can prevent conflicts from negatively affecting a research collaboration by putting pressure on the collaborators. In this vein, brokered collaboration strengthen the validity of the collaboration even though ‘equality’ and ‘reciprocity’ may not be easily discerned. Hence, the requirements of a relaxed atmosphere and friendly interpersonal relationships on a loosely-coupled research team become important when research collaborations are conducted on a voluntary basis. In terms of creating policies for voluntary research collaboration, not only should policy makers invest enough funds to sponsor various and diverse brokered projects, but they should also develop more networking channels among researchers [22]. In this way, the brokered collaboration partners may also develop a close relationships, which in turn may make those relationships tighter to serve the goodness of the organization. Also, some voluntary collaboration behaviors may be born by brokered collaboration, thus facilitating more research output even the brokered collaboration relationship was terminated.

## Limitations

There are several limitations to our study. First, in our sample, some international Chinese students studying in Flemish universities are included (N = 33). In the latent regression analysis, these students were classified according to their Chinese nationality because the majority of them are raised in Chinese culture origin even though, as international students, they were also cultivated in Flemish universities. It is possible that this classification has weakened our findings on the differences university tiers have on the networking channel prerequisite. However, with the internationalisation of Chinese higher education, researchers that have cross-border research experience are to some extent already the hallmark of Chinese researchers [83]. Second, because our study sample included student researchers from Belgium and China, more evidence is needed before the results can be generalised to researchers beyond this context. Third, since we mainly surveyed early-career researchers that have prior experience of voluntary research collaboration, it would be interesting, in future research, to study the differences between the prerequisite preferences of early-career researchers who have not yet collaborated in a research project (brokered research collaboration) and those who have. Also, since our study is an explorative one and is based on a modest sample size, factors concerning nationality, age, working experience, disciplines are only vaguely differentiated; future research might consider to expand the sample so that to identify how above-mentioned factors influence collaboration prerequisites in detail.

## Conclusion

Our study’s contributions to the current collaboration study are threefold. First, it aligns with previous literature by generalising the requirements for research collaboration and classifying them into substance- and entity-related prerequisites, as well as socially oriented ones. We discovered that, in contrast to brokered research collaboration, voluntary research collaboration relies more heavily on socially oriented prerequisites that qualifies the characteristics of potential research collaborator, such as being reciprocal, trustworthy, equal and accountable. Second, by analysing determinants such as nationality, maturity level and discipline in divergent perceptions of research collaborator prerequisites, our study has practical implications for voluntary research collaboration. Specifically, Chinese researchers and older, more experienced researchers are inclined to stress the importance of equality, and Chinese researchers emphasise the necessity of funds, while researchers from first-tier universities place more value on networking channels. Furthermore, disciplinary differences for prerequisite of proximity have also been discovered. Third, based on the research of Shrum et al. [25], we differentiated the distinctness between brokered research collaborations and voluntary ones, which could be of great use to researchers seeking collaborative partners in the future.

## Supporting information

S1 Table Latent class regression result on equality. (XLSX)

S2 Table Latent class regression result on funds and material supplies. (XLSX)

S3 Table Latent class regression result on channels of network. (XLSX)

S4 Table Latent class regression result on physical proximity. (XLSX)
